# Hormones and diet: low insulin-like growth factor-I but normal bioavailable androgens in vegan men

**DOI:** 10.1054/bjoc.2000.1152

**Published:** 2000-06-02

**Authors:** N E Allen, P N Appleby, G K Davey, T J Key

**Affiliations:** Cancer Epidemiology Unit, Imperial Cancer Research Fund, Gibson Building, Radcliffe Infirmary, Woodstock Road, Oxford, OX2 6HE, UK

**Keywords:** diet, insulin-like growth factor-I, sex hormones, vegan, meat-eaters

## Abstract

Mean serum insulin-like growth factor-I was 9% lower in 233 vegan men than in 226 meat-eaters and 237 vegetarians (*P* = 0.002). Vegans had higher testosterone levels than vegetarians and meat-eaters, but this was offset by higher sex hormone binding globulin, and there were no differences between diet groups in free testosterone, androstanediol glucuronide or luteinizing hormone. © 2000 Cancer Research Campaign


*British Journal of Cancer* (2000) **83**(1), 95–97
© 2000 Cancer Research Campaign
doi: 10.1054/ bjoc.2000.1152, available online at http://www.idealibrary.com on IDEAL®

### Hormones and diet: low insulin-like growth factor-I but normal bioavailable androgens in vegan men

**NE Allen, PN Appleby, GK Davey and TJ Key**

Cancer Epidemiology Unit, Imperial Cancer Research Fund, Gibson Building, Radcliffe Infirmary, Woodstock Road, Oxford OX2 6HE, UK

**Summary** Mean serum insulin-like growth factor-I was 9% lower in 233 vegan men than in 226 meat-eaters and 237 vegetarians ($P = 0.002$). Vegans had higher testosterone levels than vegetarians and meat-eaters, but this was offset by higher sex hormone binding globulin, and there were no differences between diet groups in free testosterone, androstanediol glucuronide or luteinizing hormone. © 2000 Cancer Research Campaign

**Keywords:** diet; insulin-like growth factor-I; sex hormones; vegan; meat-eaters

A western diet, believed to be a risk factor for prostate cancer (Giles and Ireland, 1997; Bosland et al, 1999) may increase risk by increasing androgen concentrations in the serum and prostate (Montie and Pienta, 1994). Data from prospective studies suggest that prostate cancer risk may be increased by high serum concentrations of bioavailable testosterone (Gann et al, 1996) and androstanediol glucuronide (A-diol-g), a serum marker of 5α-reductase activity and intraprostatic dihydrotestosterone (DHT) (Eaton et al, 1999). One prospective study has also found circulating levels of insulin-like growth factor-I (IGF-I), a polypeptide which interacts with androgens to stimulate cell proliferation, to be higher among men who subsequently developed prostate cancer than among controls (Chan et al, 1998).

The nutritional determinants of circulating hormone levels in men are poorly understood; some previous studies suggested that a vegetarian diet, low in saturated fat and high in dietary fibre, might reduce androgen levels, but the results have been inconsistent and based on small numbers (Deslypere and Vermeluen, 1984; Howie and Shultz, 1985; Bélanger et al, 1989; Key et al, 1990; Pusateri et al, 1990). We are not aware of any previous data on IGF-I concentrations in vegetarian and vegan men. We report here the results of a large cross-sectional study of serum concentrations of sex hormones and related proteins among 696 men of whom 226 were meat-eaters, 237 were vegetarians and 233 were vegans. We estimated that the study would have 80% power to detect 10% differences in hormone concentrations between diet groups at the 5% significance level.

### MATERIALS AND METHODS

750 white male subjects were selected from the Oxford UK component of the European Prospective Investigation into Cancer and Nutrition (EPIC), and were for the current study if they had donated a blood sample prior to 1998. Subjects were excluded if they had a self-reported history of cancer ($n = 16$), or were taking medication known to influence hormone levels ($n = 16$). We also excluded those who had insufficient serum available for analysis ($n = 16$) or had an estimated energy intake of < 3.1 and > 18.40 mJ/day ($n = 5$). One further individual was excluded due to an SHBG level above the upper detection limit of 280 nmol/l.

The present study includes 696 men of whom 226 were meat-eaters, 237 were vegetarians and 233 were vegans, recruited between 1994 and 1997. 30 ml blood samples were collected for each subject and sent in the mail to the EPIC laboratory in Norfolk and aliquoted into plasma, serum, buffy coat and erythrocytes. Serum samples were stored in liquid nitrogen tanks at –196°C. Nutrient intakes were estimated from a validated semi-quantitative food frequency questionnaire by multiplying the nutrient content of a specific portion size of each food by the frequency of consumption (Bingham et al, 1997). Details of medical history and lifestyle characteristics were also recorded.

Assays for serum IGF-I, sex hormone binding globulin (SHBG), testosterone (T), A-diol-g, and luteinizing hormone (LH) were performed in the Clinical Biochemistry Laboratory at the John Radcliffe Hospital, Oxford in 1998. Plasma cholesterol assays were performed using automated enzymatic procedures at the Biochemistry Laboratory at Kings College, London in 1999. Each assay batch included equal numbers of meat-eaters, vegetarians and vegans, selected at random. All intra-assay coefficients of variation were less than 12%. An estimate of the concentration of free testosterone (FT) was derived from the known concentrations of T and SHBG and assuming albumin concentration to be constant between individuals, using the formula based on the law of mass action (Södergaard et al, 1982).

Hormone concentrations and nutrient intakes were logarithmically or square-root transformed to approximate normal distributions. The mean hormone concentrations and their corresponding 95% confidence intervals (95% CI) are presented as back-transformed values. Mean values adjusted for covariates were calculated by analysis of covariance.

### RESULTS

Anthropometric and dietary characteristics are shown in Table 1. Vegetarians and vegans were significantly younger and had a

*Received 30 November 1999*
*Revised 7 February 2000*
*Accepted 8 February 2000*

*Correspondence to:* NE Allen

**Table 1** Anthropometric and nutritional characteristics by dietary group

| Variable | Meat-eaters ($n$ = 226) | Vegetarians ($n$ = 237) | Vegans ($n$ = 233) | $P$ value[a] |
|---|---|---|---|---|
| Age (yrs) | 52.8 (51.2–54.3) | 46.3 (44.8–47.9) | 42.9 (41.3–44.4) | < 0.0001 |
| Weight (kg) | 82.4 (80.9–83.4) | 74.4 (72.9–75.9) | 71.8 (70.4–73.3) | < 0.0001 |
| Height (m) | 1.78 (1.77–1.79) | 1.78 (1.77–1.79) | 1.78 (1.77–1.79) | 0.4739 |
| BMI[b] | 26.1 (25.7–26.5) | 23.4 (22.0–23.8) | 22.7 (22.3–23.1) | < 0.0001 |
| Energy (MJ) | 10.3 (9.91–10.7) | 8.88 (8.57–9.20) | 8.08 (7.79–8.38) | < 0.0001 |
| Protein (% energy) | 16.6 (16.3–16.8) | 13.2 (12.9–13.4) | 12.7 (12.4–13.0) | < 0.0001 |
| Total fat (% energy) | 34.0 (33.2–34.8) | 31.0 (30.3–31.8) | 29.9 (29.1–30.7) | < 0.0001 |
| Saturated fatty acids (% energy) | 11.8 (11.5–12.1) | 8.71 (8.38–9.03) | 4.87 (4.55–5.20) | < 0.0001 |
| Monounsaturated fatty acids (% energy) | 10.9 (10.5–11.2) | 8.61 (8.26–8.95) | 8.57 (8.22–8.92) | < 0.0001 |
| Polyunsaturated fatty acids (% energy) | 5.39 (5.06–5.72) | 6.12 (5.79–6.44) | 8.17 (7.85–8.50) | < 0.0001 |
| P:S ratio[c] | 0.49 (0.44–0.54) | 0.78 (0.73–0.83) | 1.71 (1.66–1.76) | < 0.0001 |
| Cholesterol (mg/d) | 327 (301–355) | 112 (108–127) | 20.8 (19.2–25.5) | < 0.0001 |
| Alcohol (% energy) | 5.83 (5.07–6.58) | 4.30 (3.57–5.03) | 3.96 (3.22–4.70) | 0.0012 |
| Non-starch polysaccharides (g/d) | 17.7 (16.9–25.8) | 23.8 (22.8–24.8) | 27.5 (26.3–28.7) | < 0.0001 |

Age and anthropometric measurements are presented as arithmetic means and corresponding 95% confidence interval (CI); nutrients were natural log-transformed and are presented as geometric means and 95% CI. [a]P value is test of heterogeneity, [b]BMI: body mass index = weight [kg]/height [m], [c]polyunsaturated:saturated fatty acid ratio.

**Table 2** Mean hormone concentrations in three dietary groups

| Hormone | Meat-eaters ($n$ = 226)[a] | Vegetarians ($n$ = 237)[a] | Vegans ($n$ = 233)[a] | $P$ value[b] |
|---|---|---|---|---|
| **IGF-I (nmol/l)** | | | | |
| Unadjusted for BMI | 20.1 (19.3–20.8) | 20.1 (19.4–20.8) | 18.5 (17.8–19.2) | 0.002 |
| Adjusted for BMI | 20.3 (19.5–21.1) | 20.0 (19.3–20.7) | 18.4 (17.7–19.1) | 0.002 |
| **SHBG (nmol/l)** | | | | |
| Unadjusted for BMI | 42.6 (40.2–45.2) | 44.6 (42.2–47.0) | 50.9 (48.1–53.7) | 0.001 |
| Adjusted for BMI | 45.7 (43.1–48.4) | 43.6 (41.4–46.0) | 48.7 (46.0–51.4) | 0.017 |
| **T (nmol/l)** | | | | |
| Unadjusted for BMI | 19.3 (18.3–20.3) | 20.5 (19.6–21.4) | 22.3 (21.3–23.4) | 0.003 |
| Adjusted for BMI | 20.3 (19.3–21.3) | 20.1 (19.2–21.0) | 21.7 (20.7–22.7) | 0.046 |
| **FT (nmol/l)** | | | | |
| Unadjusted for BMI | 0.40 (0.38–0.42) | 0.42 (0.40–0.44) | 0.42 (0.40–0.45) | 0.137 |
| Adjusted for BMI | 0.42 (0.40–0.44) | 0.43 (0.41–0.45) | 0.44 (0.42–0.46) | 0.471 |
| **A-diol-g (nmol/l)** | | | | |
| Unadjusted for BMI | 8.93 (8.40–9.47) | 8.45 (7.97–8.93) | 8.63 (8.14–9.14) | 0.443 |
| Adjusted for BMI | 8.72 (8.18–9.28) | 8.49 (8.01–8.98) | 8.80 (8.29–9.32) | 0.646 |
| **LH (IU/l)** | | | | |
| Unadjusted for BMI | 5.29 (4.91–5.65) | 5.27 (4.96–4.61) | 5.30 (4.96–5.66) | 0.996 |
| Adjusted for BMI | 5.43 (5.06–5.82) | 5.23 (4.92–5.57) | 5.20 (4.87–5.56) | 0.632 |
| **Total cholesterol (nmol/l)** | | | | |
| Unadjusted for BMI | 4.94 (4.81–5.07) | 4.55 (4.44–4.66) | 4.08 (3.97–4.19) | < 0.0001 |
| Adjusted for BMI | 4.87 (4.87–5.00) | 4.57 (4.46–4.69) | 4.12 (4.01–4.24) | < 0.0001 |

Back-transformed means presented with 95% CI in parentheses. Values are adjusted for age (in categories of 20–29, 30–39, 40–49, 50–59, 60–69, 70+), smoking status (never, past, < 10 cigarettes/day, 10+ cigarettes/day), vigorous exercise (< 2, 2–4 5+ hours/week), time of day of venipuncture (< 10, 10–13.29, 13.30+ hours), time since last meal at venipuncture (< 1.5, 1.5–3, 3+ hours) and time between blood draw and processing (1, 2, 3, 4+ days). [a]Insufficient serum led to IGF-I measurement being unavailable in 1 subject, SHBG in 9 subjects, T in 20 subjects, FT in 25 subjects, A-diol-g in 5 subjects, LH in 20 subjects and total cholesterol in 8 subjects. [b]$P$ value is test of heterogeneity.

lower weight and body mass index (BMI) than meat-eaters. Examination of nutrient intakes showed that vegetarians and vegans had lower intakes of energy, protein, total fat, saturated and monounsaturated fatty acids, and alcohol (each as percent energy) and lower intakes of dietary cholesterol compared to meat-eaters. Conversely, vegans had higher intakes of polyunsaturated fatty acids (% energy), polyunsaturated:saturated fatty acid ratio and non-starch polysaccharides than meat.

All mean hormone concentrations are adjusted for age, smoking status, vigorous exercise, time of day of venipuncture, time since last eaten at venipuncture and time between venipuncture and blood processing. Mean hormone concentrations are presented with and without adjustment for BMI (Table 2). Vegan men had on average 9% lower IGF-I levels than meat-eaters ($P$ < 0.01) and 8% lower levels than vegetarians ($P$ < 0.01); adjustment for BMI made little difference to these values. Prior to adjustment for BMI, SHBG levels in vegans were 16% higher than in meat-eaters ($P$ < 0.0001), and 12% higher than in vegetarians ($P$ = 0.0008); adjustment for BMI reduced these differences to 6% ($P$ = 0.02) and 10% ($P$ = 0.004), respectively. Vegans had 13% higher T concentration than meat-eaters ($P$ = 0.0001) and 8% higher than vegetarians ($P$ = 0.001); adjustment for BMI reduced these differences to 6% ($P$ = 0.07) and 7% ($P$ = 0.02), respectively. Since an increase in SHBG generally causes an increase in total T, we examined differences in mean T concentration after additionally adjusting for SHBG. This adjustment substantially reduced the difference in T between

© 2000 Cancer Research Campaign the three diet groups (adjusted means were 20.3, 20.5 and 21.2 nmol/l in meat-eaters, vegetarians and vegans respectively, test for heterogeneity; $P = 0.312$). There were no significant differences in calculated FT, A-diol-g or LH between dietary groups. There were substantial differences in plasma total cholesterol, with vegans having 17% lower mean values than meat-eaters ($P < 0.0001$), and 10% lower values than vegetarians ($P < 0.0001$), and adjustment for BMI made little difference to these values.

### DISCUSSION

This study is the largest to date to investigate differences in serum hormone concentrations between meat-eaters, vegetarians and vegans. The significant 9% lower IGF-I concentration among vegan men compared to meat-eaters has not been reported before. IGF-I may play an important role in the aetiology of prostate cancer via its ability to interact with androgens to stimulate prostatic cell growth (Cohen et al, 1994), but its determinants are poorly understood. Chan et al (1998) found that men who subsequently developed prostate cancer had 8% higher serum IGF-I concentrations than men who remained healthy, suggesting that the 9% difference we observed is large enough to significantly alter prostate cancer risk.

SHBG was significantly higher in the vegans than in the meat-eaters, leading to a corresponding increase in T in order to maintain constant levels of FT, a pattern which has been found in previous smaller observational studies (Key et al, 1990; Pusateri et al, 1990). The differences in SHBG concentrations between dietary groups were reduced but not eliminated by adjusting for differences in BMI, suggesting that nutritional factors specific to a vegan diet may be important determinants of circulating SHBG levels, over and above their effect on BMI.

The significantly lower plasma total cholesterol concentration found among vegans compared to both vegetarians and meat-eaters, and the lower concentration in vegetarians compared to meat-eaters has been well-established in previous observational studies (Thorogood et al, 1987). These results confirm that large differences in lipid intake and lipoprotein physiology do exist between these dietary groups, but that these differences are not associated with circulating androgen concentrations.

The results did not support the hypothesis that meat-eaters have higher levels of bioavailable androgens than non meat-eaters. No differences in hormone levels were found between meat-eaters and lacto-ovo-vegetarians, suggesting that vegetarian diets may not alter prostate cancer risk, but the relatively low IGF-I levels in vegans might reduce their risk of prostate cancer. Prospective data have shown that vegetarians do not have significantly lower prostate cancer mortality rates than comparable non-vegetarians (Key et al, 1999), but these subjects were predominantly lacto-ovo-vegetarians and there are, as yet, no data on prostate cancer rates among vegans.

### ACKNOWLEDGEMENTS

We thank all the participants of the EPIC study, the EPIC study staff at ICRF, Dr Dennis Quantrill and staff at the Clinical Biochemistry laboratory at the John Radcliffe Infirmary, Oxford, and TAB Sanders and Z Lloyd-Wright at the Nutrition and Dietetics Department at Kings College, London. This study was supported by the Imperial Cancer Research Fund and by the Europe Against Cancer Programme of the Commission of the European Communities.

### REFERENCES

Bélanger A, Locong A, Noel C, Cusan L, Dupont A, Prévost J, Caron S and Sévigny J (1989) Influence of diet on plasma steroid and sex plasma binding globulin levels in adult men. *J Steroid Biochem* **32**: 829–833

Bingham SA, Gill C, Welch A, Cassidy A, Runswick SA, Oakes S, Lubin R, Thurnham DI, Key TJ, Roe L, Khaw KT and Day NE (1997) Validation of dietary assessment methods in the UK arm of EPIC using weighed records, and 24-hour urinary nitrogen and potassium and serum vitamin C and carotenoids as biomarkers. *Int J Epidemiol* **26** (Suppl 1): S137–S151

Bosland MC, Oakley-Girvan L and Whittemore AS (1999) Dietary fat, calories and prostate cancer risk. *J Natl Cancer Inst* **91**: 489–491

Chan JM, Stampfer MJ, Giovannucci E, Gann PH, Wilkinson P, Hennekens CH and Pollak M (1998) Plasma insulin-like growth factor-I and prostate cancer risk: a prospective study. *Science* **279**: 563–566

Cohen P, Peehl DM and Rosenfeld RG (1994) IGF axis in the prostate. *Horm Metab Res* **26**: 81–84

Deslypere JP and Vermeulen A (1984) Leydig cell function in normal men: effect of age, lifestyle, residence, diet and activity. *J Clin Endocrinol Metab* **59**: 955–962

Eaton NE, Reeves GK, Appleby PN and Key TJ (1999) Endogenous sex hormones and prostate cancer: a quantitative review of prospective studies. *Br J Cancer* **80**: 930–934

Gann PH, Hennekens CH, Ma J, Longcope C and Stampfer MJ (1996) Prospective study of sex hormone levels and risk of prostate cancer. *J Natl Cancer Inst* **88**: 1118–1126

Giles G and Ireland P (1997) Diet, nutrition and prostate cancer. *Int J Cancer* (Suppl 10): S13–S17

Howie BJ and Shultz TD (1985) Dietary and hormonal interrelationships among vegetarian Seventh Day Adventists and nonvegetarian men. *Am J Clin Nutr* **42**: 127–134

Key TJ, Roe L, Thorogood M, Moore JW, Clark GM and Wang DY (1990) Testosterone, sex hormone binding globulin, calculated free testosterone and oestradiol in male vegans and omnivores. *Br J Nutr* **64**: 111–119

Key TJ, Fraser GE, Thorogood M, Appleby PN, Beral V, Reeves G, Burr ML, Chang-Claude J, Frentzel-Beyme R, Kuzma JW, Mann J and McPherson K (1999). Mortality in vegetarians and non-vegetarians: detailed findings from a collaborative analysis of 5 prospective studies. *Am J Clin Nutr* **70** (Suppl): 516S–524S

Montie JE and Pienta KJ (1994) A review of the role of androgenic hormones in the pathogenesis of benign prostatic hyperplasia and prostate cancer. *Urology* **43**: 892–899

Pusateri DJ, Roth WT, Ross JK and Shultz TD (1990) Dietary and hormonal evaluation of men at different risks for prostate cancer: plasma and fecal hormone–nutrient interrelationships. *Am J Clin Nutr* **51**: 371–377

Södergaard RR, Backstrom T, Shanbhag V and Carstensen H (1982) Calculation of free and unbound fractions of testosterone and estradiol-17β to human plasma proteins at body temperature. *J Steroid Biochem* **6**: 801–810

Thorogood M, Carter R, Benfield L, McPherson K and Mann JI (1987) Plasma lipids and lipoprotein cholesterol concentrations in people with different diets in Britain. *BMJ* **295**: 351–353

*© 2000 Cancer Research Campaign*
